# The role of social accountability in changing service users’ values, attitudes, and interactions with the health services: a pre-post study

**DOI:** 10.1186/s12913-023-09971-x

**Published:** 2023-09-06

**Authors:** Victoria Boydell, Petrus S. Steyn, Joanna Paula Cordero, Ndema Habib, My Huong Nguyen, Dela Nai, Donat Shamba, Kamil Fuseini, Sigilbert Mrema, James Kiarie

**Affiliations:** 1 Institute of Women’s Health, University College London, London, UK; 2 UNDP/UNFPA/UNICEF/WHO/World Bank Special Programme of Research, Development and Research Training in Human Reproduction, Avenue Appia 20, Geneva, 1202 Switzerland; 3 Population Council, 204 Yiyiwa Drive, Abelemkpe, Accra, Ghana; 4 Department of Health Systems, Impact Evaluation and Policy, Ifakara Health Institute, P.O.BOX 78373, Dar es Salaam, Tanzania

**Keywords:** Contraception, Social accountability, Attitudes, Ghana, Tanzania

## Abstract

This study evaluated the effects of community engagement through social accountability on service users’ values, attitudes and interactions. We conducted a pre–post study of the community and provider driven social accountability intervention (CaPSAI) over a 12-month period among 1,500 service users in 8 health facilites in Ghana and in Tanzania (n = 3,000).

In both countries, there were significant improvements in women’s participation in household decision-making and in how service users’ perceive their treatment by health workers. In both settings, however, there was a decline in women’s knowledge of rights, perception of service quality, awareness of accountability mechanisms and collective efficacy in the community. Though CaPSAI intervention set out to change the values, attitudes, and interactions between community members and those providing contraceptive services, there were changes in different directions that require closer examination.

## Introduction

In recent years there has been much emphasis on strengthening the relationship between health system actors and community members. One such tool is social accountability, defined here as “*citizens’ efforts at ongoing meaningful collective engagement with public institutions for accountability in the provision of public goods (*p. 161) [1].” Scorecards, social audits, and participatory budgeting, popular social accountability tools, are used to facilitate processes that creates opportunities and spaces for those seeking services and those responsible for providing them to come together and mutually identify barriers and solutions to improve their services. Joy Moncrieffe (2011) has argued that social accountability is best understood as a relational process in which people’s values, attitudes, and interactions change as a consequence of participating [2]. These changes in respective norms, values, attitudes, and relationships toward each other and themselves as a result of this dialectic interaction has been borne out in the evidence [3, 4].

Recognizing the importance of values and attitudes on the delivery and performance of the health system is by no means new. We can see how values and attitudes affect health seeking behaviour - in the way community social norms surround women’s decisions about health care [5], or in how providers’ bias and beliefs about a service user affect how they counsel and treat them [6]. Moreover, the quality of people’s interactions also affects the performance of the healthcare systems as reflected in the respectful care movement [7], and more recently in health system responsive literature [8]. These trends are part of the increasing recognition of “soft skills” or the “software” of the health system, that is the values, norms, attitude, communication skills and collaboration practices at work [9]. Others regard the performance of the health system as the product of interactions between system ‘hardware’ (such as infrastructure, medicines and workforce) and the ‘software’ (the more latent components such as human values, power dynamics and norms) [10].

Supporting social accountability processes is one of several ways to change values and attitudes of both those using and those providing services. The affective effects of social accountability has been widely documented and captured as governance related outcomes such as increased community participation [3, 11, 12], and increased confidence among women to claim rights and make demands [13–15]. Other relevant changes include the responsiveness of duty-bearers [8, 16], and their increased awareness of community needs [15, 17, 18]. In addition, the type and quality of interactions between duty-bearers and those claiming their rights has been acknowledged in the increased community engagement in decision-making [3, 4], more meaningful provider-service user interaction [3, 4], and in enhanced mutuality and trust [15, 19–21]. Yet these important changes in service users and providers’ values and attitudes are often not measured because they pose several methodological challenges [18, 22].

One notable exception is CARE’s Women’s Voice tool, a validated psychometric measure that captures these variables and provides an innovative starting point to capturing values, attitudes, and interactions [23]. As part of a more extensive complex intervention study on social accountability in the context of contraceptive services [24], the authors of this paper first adapted and validated CARE’s measures of service users’ attitudes and behaviours [25], and then used the measures to understand the processes by which change was affected. In the Community and Provider driven Social Accountability (CaPSAI) Project’s theory of change (ToC) these values, attitudes and interactions are set as intermediate outcomes along the casual pathway and are directly influenced by the social accountability process under study. In this paper, we share the findings about how service users’ values, attitudes, and interactions changed as a result of a social accountability (SA) initiative in Ghana and Tanzania.

## Methods

### Study design

This study was a quasi-experimental, pre-post evaluation cross sectional study of service user’s values, attitudes, and interactions as part of the larger Community and Provider driven Social Accountability (CaPSAI) Project undertaken in Ghana and Tanzania. [24]. The CaPSAI Project contributes to the evidence on the effects of social accountability and participatory processes in the context of a family planning and contraceptive programmes. The study was designed according to the Medical Research Council (MRC) guidance on complex interventions and was based on a theory of change using a co-designed intervention to account for the complexity of SA processes [24, 26–28]. This study accounted for the multiple components required to track the different levels and interrelated outcomes, including changes in values, attitudes and interactions that this paper focuses on [24].

**Fig. 1:**
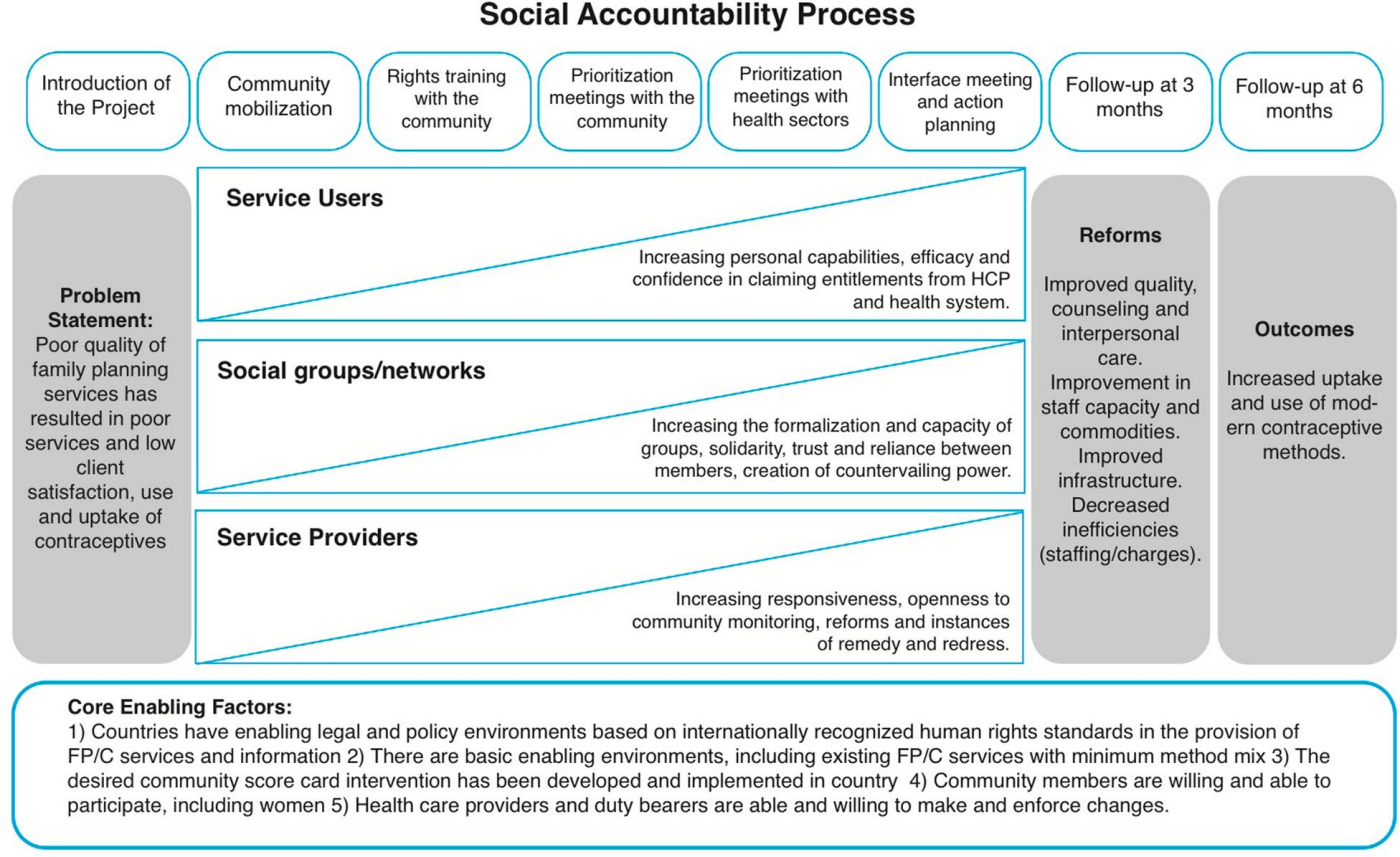
Taken from [24]:

The constructs assessed in this study are drawn from the CaPSAI Theory of Change in Fig. 1, these are the intermediate outcomes of expanding inclusive and effective negotiated spaces, empowered health workers, and empowered women and community members [24, 25]. The theory of change was developed to link the intervention components (at the top of the figure with the intermediate) and the intermediate and distal outcomes (on the righthand side of the figure). The development and validation of the measures used in this pre–post evaluation study have been outlined elsewhere [25]. This study aimed to (1) gauge change in the validated items at pre- and post-the intervention, and (2) measure intermediate outcomes.

The CaPSAI intervention is a co-designed social accountability process that is responsive to local contexts and practices [26, 27]. In the community surrounding a facility, community members and health workers were separately trained by civil society organisations in their rights and entitlements and each group is then taken through a process by the civil society organisations to generate issues and then rank what they consider to be a priority. The civil society organisation leading the process then bring together the two groups, community members and health workers, in a facilitated interface meeting to jointly identify shared issues and solutions to address them. The community members and health workers together then met with local government officials to discuss their concerns and priorities and make action plans to respond to the claims made. These action plans were then followed up and reported on over a 6-month period.

The social accountability process was composed of nine steps led by a national civil society partner over a six-month period. Each step and its purpose are outlined in Table 1. A full description the intervention has published in the WHO (2021) *Community and provider-driven social accountability intervention for family planning and contraceptive service provision: experiences from the field* [27].

**Table 1:** Steps of the social accountability intervention [27]

Step	Title	Purpose and description
Step one	Preparatory work	To prepare the necessary institutional and social permissions and approvals required to implement the social accountability process in a community.
Step two	Introduction to local authorities and gatekeepers	To ensure that local authorities and gatekeepers are both aware and supportive of the process.
Step three	Mobilization and introduction of the project to the community members	To spread awareness of the project among the local community and ensure inclusive participation from a wide range of community members.
Step four	Health, rights and civic education with community members	To share information about sexual and reproductive health and rights (SRHR) and entitlements with community members. To work together to explore any perceived gaps or shortcomings in the services they receive. To generate discussion on the local issues that faced by the population.
Step five	Health, rights and standards of care sensitization with health actors	To share information about srhr and entitlements with health providers. To work together to explore any perceived gaps or shortcomings in the services they receive. To generate discussion on key issues faced by local people.
Step six	Prioritization meeting with the community	To ensure that a diverse range of community members identify and rank the most pressing issues related to fp/c information and services in their community.
Step seven	Prioritization with health actors	To ensure that health care providers identify and rank the most pressing issues related to fp/c information and services in their community.
Step eight	Interface meeting and joint action planning	To share the assessments separately generated by community and health actors and then to jointly identify areas for improvement. To develop an action plan to ensure concrete measures are taken to improve services and/or maintain good practices.
Step nine	Regular ongoing monitoring and follow-up	To track if progress has been made in the jointly- agreed action plan, by regularly following up with both the community and health authorities. To present an opportunity to involve high-level duty bearers or third parties in addressing unresolved issues.

### Participant selection and enrolment

Possible study participants were identified in eight intervention facilities in each country during consultations by health providers who referred them to study staff. Screening was done by the study staff using the eligibility criteria that included age (15–49) who were attending the FP services of health facilities involved in a participating study facility, were first-time or continuing users contraception and provided consent to participate. Following the consent or assent process, the interviews were conducted in person at the facility, or an appointment was set later. The same eligibility criteria for participants were used in all intervention sites.

### Data collection

A sample of over 750 women aged 15 to 49 years accessing contraceptive services was interviewed prior to the start of the intervention and an additional 750 women aged 15 to 49 years after the intervention in each country. Sampling for the service users survey was calculated using *a priori* sample size calculation with the ratio of ten subjects per item ratio and guidance of more than 500 which equals a very good sample for validation [25, 26, 29, 30]. The calculation was based on 75 items. The pre- and post-survey were taken eight months apart.

A total of 118 questions were asked of respondents upon leaving a facility, and only 44 scale items and 11 domains were included in the following analysis. The other items included questions about demographic status, reproductive and family planning history, relationship status, income, occupation, and religion. The 11 domains measured were driven by the theory of change, which was informed by existing empirical and theoretical work on social accountability. After defining the domains, we identified existing validated measures for each domain. We drew on CARE’s Women VOICES tool, a validated measure the aimed to capture similar intermediate outcomes concerning maternal health in Malawi [23]. In addition, we included three domains to represent the CaPSAI theory of change – namely ‘knowledge and awareness of rights, self-efficacy and political capabilities. The items were validated through consultations with experts and with World Health Organization (WHO) Forms Committee. The instruments were psychometrically tested at baseline in Ghana and Tanzania and the findings were published [23]. Table 2 outlines the domains that were included in the final analysis.

**Table 2:** Domains measured based on theory of change [23]

Domains	Number of Items
Knowledge of Health Rights++	6 **
Women’s participation in household decision- making*	10**
Self-efficacy with health care providers ++	3**
Perception of service quality *	3**
Mistreatment by Health workers ++	4**
Collective efficacy *	4**
Community support in time of crisis *	4**
Mutual responsibility for and support of services*	5**
Awareness of accountabilty mechanisms++	3**
Ability to participate in community meetings*	3**
Ability to atttend community meetings*	3**

* validated from CARE’s Women VOICES Tool

++ new scales

** scales tested in this study

Post-intervention survey was conducted at the facility in a private location. In Ghana, a total of 15 data collectors (5 females and 10 males for the first survey; 8 females and 7 males for the second survey) were trained over a 3-day training workshop from 4 to 6 April 2018 and a 2-day refresher from July 9–10 2019, respectively. In Tanzania, a total of 15 data collectors (9 females and 6 males) were trained over the survey over a period of 5 days from 19 to 23 March in 2018 and a 5-day refresher training was conducted from 29 to 2019 to 2 August 2019. Data collection was conducted using a tablet-based questionnaire to capture real-time data using OpenClinica and was later uploaded onto a secure server. In Tanzania, the first survey data collection started on 26 March and was completed on 25 May 2018. The second survey took place from 2 to 2019 to 3 October 2019. All respondents chose to be interviewed in Kiswahili. In Ghana, data collection for the first survey started on 9 April 2018 and was completed on 4 June 2018, and 46.4% chose to be interviewed in English, while 53.6% chose to be interviewed in Akan. The second survey took place between 10 and 2019 and 10 October 2019, and 54.5% chose to be interviewed in English, while 45.5% chose to be interviewed in Akan.

### Patient and public involvement

It was not appropriate or possible to involve patients or the public in the design, or conduct, or reporting, or dissemination plans of our research.

### Ethical consideration

Once assessed as eligible, respondents completed the informed consent process. There were no incentives given to women and girls to participate in the study. However, study participants who agreed to participate were reimbursed for their travel cost, where it was permitted by country-specific ethical requirements. In Ghana, the research team supported the travel cost to the facility with five Ghana cedis (~ 1 US dollar) given after the interview. In Tanzania, no reimbursements were given.

For adolescent participants (15–17 years) identified to participate in the study, research staff explained what the study is about and the requirements for their participation in the study, including obtaining parental consent. If the adolescent approves, parental/guardian consent was sought and once done, the participant was also guided through the assent process. Emancipated adolescents were able to provide consent in both countries.

CaPSAI Project master and country protocols (Project ID A65896) were approved by technical and ethics review committees at the World Health Organization (WHO). Additionally, the country protocols were reviewed and approved by the Population Council Institutional Review Board (exemption approval - # EX201714) and Ghana Health Service Ethics Review Committee (GHS-ERC:009/08/2017) in Ghana. In Tanzania, the protocol has been approved by Ifakara Health Institute Institutional Review Board (IHI/IRB/No:18-2018 and IHI/IRB/AMM/No:03-2019) and the National Institute of Medical Research (NIMR) review board (NIMR/HQ/R.8a/Vol.IX/2668), as well as the NIMR/Mbeya Medical Research and Ethics Review Committee (GB.152/377/01/214a).

### Statistical analysis

To detect statistically significant differences in the distribution of sociodemographic characteristics between the groups at pre-intervention and post-intervention in each country, we used a chi-square or a Fisher’s exact test for categorical variables; and a t-test for comparison of continuous variables. For comparison of continuous pre- and post-intervention intermediate outcomes a t-test was used. We conducted a two-sided test, with type 1 error at 5% level. Statistical Analysis System (SAS) Version 9.4 was used for the statistical analyses.

## Results

### Demographic characteristics

The demographic characteristics of the study population were similar in the pre- and post-intervention samples, see Table 3 for each country. In Ghana, most women were 21 to 35 years of age, 70.1% in the pre-group and 72.5% in the post-group, respectively. In Tanzania, the 21 to 35 age group also made up the majority of the study population, 75.6% in the pre-group and 75.7% in post-group, respectively. There were significant differences in the percentage of women who have completed primary education in Ghana, with 25.9% in the pre-group and 14.4% in the post-group, respectively. There was no difference in the percentage of the study population who had completed secondary education, 9.2% in the pre-group and 10.8% in the post-group, respectively. There were no differences in the percentage of women who have completed primary education in Tanzania, with 56.2% in the pre-group and 53.2% in the post-group, respectively. There was no difference in the percentage of the study population who had completed secondary education, 20.9% in the pre-group and 22.6% in the post-group, respectively. In both Ghana and Tanzania, there were significant differences in marital status between the pre- and post-intervention groups. In Ghana, there was a significant difference in the percentage of injectable users in the pre- and post-group. In Tanzania, there was a significant difference in the percentage of male condom users, standard days method, Lactational Amenorrhea Method, rhythm method and withdrawal in the pre- and post-group.

**Table 3:** Socio-Economic characteristics of pre- and post-groups in Ghana and Tanzania

SOCIO-DEMOGRAPHICS	Ghana				Tanzania		
	Pre-intervention		Post-Intervention	p-value	Pre-intervention	Post-Intervention	p-value
		**Age, years**					
**Mean (SD) [Min, Max]**	28.4 (7.1) [15, 49]		28.3 (7.1) (17, 49)	0.77	27.8 (6.3) [16, 47]	27.8 (6.4) [15., 49]	0.87
**Median (IR)**	27 (23, 33)		27.5 (23, 33)		27 (23, 32)	27 (23, 32)	
**≤ 20**	94 (12.5)		89 /750 (11.9)		83 (11.1)	97/752 (12.9)	
**≤ 25**	216 (28.8)		210 /750 (28.0)		231 (30.8)	217/752 (28.9)	
**≤ 30**	184 (24.5)		189 /750 (25.2)		211 (28.1)	207/752 (27.5)	
**≤ 35**	126 (16.8)		145 /750 (19.3)		125 (16.7)	145/752 (19.3)	
**≤ 40**	83 (11.1)		75 /750 (10.0)		75 (10.0)	56/752 (7.4)	
**> 40**	47 (6.3)		42 /750 (5.6)		25 (3.3)	30/752 (4.0)	
**Current contraception use**							
**IUD**	8/636 (1.3)		8/678 (1.2)	0.90	40/728 (5.5)	30/745 (4.0)	0.19
**Injectables**	458/636 (72.0)		552/678 (81.4)	< 0.0001	391/728 (53.7)	349/745 (46.9)	0.008
**Implants**	130/636 (20.4)		103/678 (15.2)	0.0128	218/728 (30.0)	175/745 (23.5)	0.005
**Pill**	33/636 (5.0)		22/678 (3.2)	0.079	122/728 (16.8)	171/745 (23.0)	0.003
**Male condom**	17/636 (2.7)		5/678 (0.7)	0.0063	53/728 (7.3)	18/745 2.4)	< 0.0001
**Female condom**	5/636 (0.8)		7/678 (1.0)	0.64	9/728 (1.2)	4/745 (0.5)	0.15
**Emergency contraception**	11/636 (1.7)		16/678 (2.4)	0.42	1/728 (0.1)	2/745 (0.3)	0.58
**Standard days method**	7/636 (1.1)		4/678 (0.6)	0.24	23/728 (3.2)	2/745 (0.3)	< 0.0001
**Lactational amenorrhea method**	6/636 (0.9)		1/678 (0.1)	0.052	27/728 (3.7)	0/745 (0.0)	< 0.0001
**Rhythm method**	4/636 (0.6)		2/678 (0.3)	0.31	50/728 (6.9)	0/745 (0.0)	< 0.0001
**Withdrawal**	18/636 (2.8)		7/678 (1.0)	0.014	23/728 (3.2)	0/745 (0.0)	< 0.0001
**Other method**	0/636 (0.2)		0/678 (0.0)	0.90	0/728 (0.0)	0/745 (0.0)	-
**Highest level of school completed**							
				< 0.0001			0.69
**Primary or lower**	568/750 (75.7		400 /750 (53.3)		500/750 (66.7)	494/752 (65.7)	
**Higher than primary education**	182/750 (24.3)		350/750 (46.7)		250/750 (33.3)	258/752 (34.3)	
**Marital status**							
				< 0.0001			< 0.0001
**Currently married**	476 (63.5)		430 /750 (57.3)		636 (84.8)	537/752 (71.4)	
**Never married**	225 (30.0)		298 /750 (39.7)		77 (10.3)	196/752 (26.1)	
**Widowed**	4 (0.5)		2 /750 (0.2)		6 (0.8)	2/752 (0.3)	
**Separated**	21 (2.8)		13 /750 (1.7)		22 (2.9)	13/752 (1.7)	
**Divorced**	20 (2.7)		7 /750 (0.9)		8 (1.1)	4/752 (0.5)	
**Other (Cohabitation, Fiancé, no husband)**	4 (0.5)		-		1 (0.1)	537/752 (71.4)	

### Changes in service user’s values, attitudes, and interactions in Ghana

In Ghana, there were several statistically significant (P < 0.05) changes found, see Table 4. There was an increase in the women’s participation in household decision-making after the intervention and there was a positive change in how people perceived they were treated by the providers. However, the service users’ knowledge of health rights declined, as did their perception of quality. Service users also noted a decline in one’s ability to attend and to participate in community meetings. There were highly significant declines (P < 0.001) in awareness of accountability mechanisms among respondents and in the collective efficacy found in the community over the course of the study.

**Table 4:** Changes in service users’ values, attitudes and interactions in Ghana

	PRE (n = 750)		POST (n = 750)		
	Mean	SD	Mean	SD	P-Value (T-test)
**Women’s participation in household decision-making**	1.77	0.33	1.82	0.31	0.0047
**Mistreatment by health workers a (self-effacy with health care providers)**	0.93	0.20	0.93	0.18	0.63
**Mistreatment by health workers b (Mistreatment by health workers)**	2.02	0.78	1.89	0.83	0.0028
**Knowledge of Health Rights**	1.65	0.50	1.57	0.49	0.0023
**Perception of service quality**	1.54	0.46	1.49	0.48	0.024
**Awareness of Accountability Mechanism**	2.40	0.88	2.08	0.72	< 0.0001
**Collective efficacy in community**	1.46	0.64	1.31	0.55	< 0.0001
**Ability to attend community meetings**	2.55	1.49	2.31	1.49	0.0017
**Ability to participate in community meetings**	2.03	1.12	2.00	1.35	0.60
**Mutual responsibility for and support of services**	1.51	0.43	1.51	0.48	0.96
**Community support in time of crisis**	1.49	0.69	1.50	0.74	0.68

### Changes in service users values, attitudes and interactions in Tanzania

In Tanzania, there were several statistically significant (P < 0.05) changes, see Table 5. Over the course of the study, women’s participation in household decision-making was also seen to improve. Service users also perceived that they were treated better by health workers. There were also improvements in the ability to attend and participate in community meetings and in mutual responsibility for and support of services. However, women’s knowledge of their health rights, the perception of service quality, their knowledge of accountability mechanisms to make claims and the sense of collective action to bring about change were all seen to decline over the study period.

**Table 5:** Changes in service users’ values, attitudes and interactions in Tanzania

	PRE (n = 750)		POST (n = 752)		
	Mean	SD	Mean	SD	P-Value (T-test)
**Women’s participation in household decision-making**	1.78	0.31	1.93	0.17	< 0.0001
**Mistreatment by health workers a (self-effacy with health care providers)**	NS	NA	NA	NA	NA
**Mistreatment by health workers b (Mistreatment by health workers)**	1.92	0.71	1.8	0.64	0.0004
**Knowledge of Health Rights**	1.59	0.48	1.49	0.46	< 0.0001
**Perception of service quality**	1.64	0.52	1.43	0.46	< 0.0001
**Awareness of Accountability Mechanism**	2.04	0.83	1.77	0.72	< 0.0001
**Collective efficacy in community**	1.87	0.96	1.73	0.84	0.0039
**Ability to attend community meetings**	2.00	1.12	2.13	1.18	0.029
**Ability to participate in community meetings**	2.38	1.26	2.63	1.37	0.0002
**Mutual responsibility for and support of services**	1.42	0.41	1.46	0.41	0.04
**Community support in time of crisis**	1.91	0.93	1.68	0.80	< 0.0001

### Comparing the findings across the two settings

Table 6 compares the findings across the two settings. In both settings, there were significant improvements in women’s participation and in household decision-making perceived treatment by health workers. In both settings there was a decline in women’s right knowledge, the perception of service quality, awareness of accountability mechanisms and collective efficacy in community. The results for several of the constructs were different between the two countries, namely the ability to attend and participate in community meetings, mutual responsibility for and support of services and community support in time of crisis.

**Table 6:** Comparing the findings across the two settings

	Ghana	Tanzania
**Women’s participation in household decision-making**	**+**	**+**
**Mistreatment by health workers**	**NS**	**NS**
**Knowledge of Health Rights**	**-**	**-**
**Perception of service quality**	**NS**	**-**
**Awareness of Accountability Mechanism**	**-**	**-**
**Collective efficacy in community**	**-**	**-**
**Ability to attend community meetings**	**-**	**NS**
**Ability to participate in community meetings**	**-**	**+**
**Mutual responsibility for and support of services**	**-**	**NS**
**Community support in time of crisis**	**+**	**-**

NS (Not significant)

## Discussion

The CaPSAI theory of change suggests that changes in the values, attitudes and interactions of the community and health providers are central part of the social accountability process leading to health-related outcomes. When comparing the results within and between countries, there are mixed results. There were only two domains where there were significant positive changes, and in five domains there were significant negative changes in the study period. This is different to the study in Malawi that used similar intermediate outcomes and reported changes in 7 out of the 13 constructs assessed [31]. There were a higher number of positive changes in Malawi compared to the findings of this study. In Malawi, there were 3–4 cycles of the social accountability process completed by the endline, whereas in the present study, only one cycle of the social accountability process was completed over a six-month period and, therefore, there was a lower level of exposure.

Bearing in mind the shorter time and limited exposure of the CaPSAI intervention, there are two significant positive trends across both countries, namely increased participation of women in household decision-making and decreased perceived mistreatment by health workers when visiting the contraceptive services, though this was not significant. These findings align with related research in this area, it is well-established that community group engagement can improve women’s decision-making power and consequently, women’s ability to make and act on decisions is linked to contraceptive use [32, 33]. There is also evidence that women’s self-help groups have had positive impacts on women’s empowerment, social support, health service use and outcomes [34, 35]. In addition, in both sites, there was a significant decrease in the mistreatment by health workers perceived by service users, service users reported less disrespect and abuse. This suggests that the intervention is having an effect on some of the attitudes and interactions of both service users and services providers in a relatively short period of time.

There were more significant positive changes in the Tanzanian context. In each country, the national partners adapted the eight-step process (which they helped co-design) to respond to the local context and practices [26]. This resulted in social accountability interventions taking different forms but retaining conceptual fidelity [26]. In the Tanzania model, nominated community members were trained in social accountability and remained present and active throughout the intervention period. The trained community members led the monitoring teams alongside the health staff and local health authorities to assess progress against the action plan, reporting back to both the communities who elected them as well as the local authorities who had been engaged. This may have created a more localised and sustained social accountability process that can be seen to positively affect the more interaction-related constructs.

We also found negative results on several of the main outcomes, namely knowledge of health rights, quality of services, awareness of accountability mechanisms, and collective efficacy. Several possible explanations can account for this. Interpreting these results is complex and does not necessarily mean an adverse outcome. One explanation of these negative/ insignificant effects on some of the outcomes at post intervention could be attributed to either selection bias as observed through differential distribution of some outcomes between women (e.g., age and education) studied at these two time points or possible intervention effects. We did not account for confounding variables in a formal regression model, and this limits our ability to explain some of these results. Another possible explanation of these results is that there has been a failure of the intervention, for example, the group at the facility that was sampled were not exposed to the intervention or there were conflicting interests between individuals that discourage joint action and collective efficacy. This could be a limitation in our theory of change, these intermediate outcomes may take a longer time to build up and could not be achieved with one cycle of the intervention.

This study had several limitations. Firstly, the intervention occurred at the community level to affect change at the facility, and the measures were taken at the facility. With this sampling strategy, it is not possible to capture those participants who attended other facilities not sampled, particularly private sector providers. Research has clearly shown the extensive role of the private sector in providing family planning and 35% of women are using private sector services in the Sub-Saharan Africa [36]. Secondly, some of the constructs examined relate to long-entrenched power relationships that can require a longer timeframe to observe changes [31]. It is worth repeating that this study could have benefitted from at least an additional cycle before the endline. Finally, there are limitations of a pre/post design, especially given that some characteristics were different between pre- and post-groups. With this study design, it is not possible to assess if the differences at endline could be attributable to selection bias or systematic differences between the groups (and not the intervention). This possibility cannot be ruled out.

## Conclusion

The CaPSAI intervention set out to change the values, attitudes and interactions between the community and those providing contraceptive services. Sugh outcomes related to knowledge (e.g. knowledge of health rights, awareness of accountability mechanism), whereas others related to attitudes (e.g. perception of service quality, collective efficacy in community, ability to attend community meetings and ability to participate in community meetings, mutual responsibility for and support of services) and others were experiences (e.g. women’s participation in household decision-making, mistreatment by health workers and community support in time of crisis). There were changes in different directions, and this suggest that we need to further examine how changes take hold and bring about certain outcomes in different timeframes.

## Data Availability

The data that support the findings of this study are available from the World Health Organisation but restrictions apply to the availability of these data, which were used under license for the current study, and so are not publicly available. Data are however available from the authors upon reasonable request and with permission of the World Health Organisation. Please contact Petrus Steyn: steynp@who.int.
